# A new species of *Anabarhynchus* (Diptera: Therevidae) from an ocean beach in south east Victoria

**DOI:** 10.3897/BDJ.2.e4016

**Published:** 2014-09-30

**Authors:** David J. Ferguson, David K. Yeates

**Affiliations:** † Australian National Insect Collection, CSIRO National Facilities & Collections, Canberra, Australia

**Keywords:** Taxonomy, diversity, therevine, beach flies, beach ecology

## Abstract

*Anabarhynchus* Macquart 1848 is a large genus of the Therevidae (Diptera) that are endemic to Australasia with a couple of described species from Melanesia. We describe and illustrate *Anabarhynchus oceanus* **sp. n.**, a species found on ocean beaches in eastern Victoria, Australia. The species shares most characters with the monobasic *Anabarhynchus kampmeierae* species group of Lyneborg (2001), but also shares a unique feature of the male genitalia with the endemic New Zealand genus *Megathereva* Lyneborg, 1992. This new species brings the total number of described Australian species in the genus to 113.

## Introduction

*Anabarhynchus* Macquart 1848 is a large genus of therevine, with over 110 species described from Australia. *Anabarhynchus* is placed in the subfamily Therevinae and is distinguished from other therevine by the presence of the following combination of characters: antennal base positioned low on frons; medial surface of scape without setae; wing cell M_3_ open (never stalked); costal setae in 2-4 rows; fore femoral macrosetae usually present; hind femora with *av* macrosetae (rarely absent); post spiracular pile present (rarely absent); mid coxa lacking pile on posterior surface (rarely present); multiple rows of post ocular macrosetae in both sexes; male frons typically broad, rarely narrower than width of ocellar tubercle and never holoptic; male genitalia typically with rounded gonocoxite usually lacking outer gonocoxal processes, inner gonocoxal apodeme present and articulated.

Australia has 112 described species in the genus *Anabarhynchus* (Lyneborg 2001, Ferguson et al. 2013, Ferguson et al. 2014) and New Zealand has 62 species of *Anabarhynchus* and three species of the closely related genus *Megathereva* Lyneborg 1992. The subspecies *Anabarhynchus hyalipennis varicincta* Bigot (1860) is found in New Caledonia and Vanuatu in the New Hebrides (Lyneborg 2001), and the genus is also known from New Guinea (Lyneborg 2001, Winterton 2011). The only other Therevine genus known to occur in the Indonesian archipelago and Papua New Guinea is *Irwiniella* Lyneborg 1976, a genus which is diverse in both the Afrotropical and Oriental regions (Winterton 2011, Nagatomi and Lyneborg 1987). *Peralia* Malloch 1932 and *Microthereva* Malloch 1932 from Chile and Argentina are closely related to *Anabarhynchus* and together form the *Anabarhynchus* genus-group which is sister to the rest of the subfamily (Webb 2006, Lambkin et al. 2009).

Throughout the last decade intensive sampling of Therevidae has been conducted in many geographical regions of Australia and has revealed that the true species diversity of *Anabarhynchus* is much higher than that described by Lyneborg (2001). It has become increasingly apparent to us that our understanding of Australian *Anabarhynchus* is still preliminary, and a more robust alignment of species into groups within the genus remains a future goal. A systematic reassessment of Lyneborg’s species groups is of a high priority.

Lyneborg revised the Australian *Anabarhynchus* (Lyneborg 2001) recognising 25 species-groups based on some external similarities, though primarily on internal morphology of both the male and female genitalia. We also indicated where *Anabarhynchus oceanus* sp. n. falls in the key by Lyneborg 2001. In the comments we discuss morphological characters that link *Anabarhynchus oceanus* sp. n. and the *Anabarhynchus kampmeierae* species-group, so as to aid in identification. This species is a large and distinctive one, and males have a characteristically modified subepandrial sclerite that may also link the species to *Megathereva* Lyneborg, 1992 from New Zealand.

 *Anabarhynchus oceanus* sp. n. brings the total number of described Australian *Anabarhynchus* to 113.

## Materials and methods

Morphological terminology used in this paper follows Mc Alpine (1981) as modified by Winterton et al. (1999) and Winterton (2006). Terms mesonotum and sternopleuron used by Lyneborg (2001) are replaced with the terms scutum and katepisternum, respectively, throughout.

Descriptions are based on the holotype, and variations within the species are provided in a section of the descriptive text titled ‘variation’, including differences found in the non-holotype sex. Frons width is determined by the width of the frons (*fw*, Fig. 1a) directly in front of the anterior ocellus, divided by the width of the anterior ocellus (*ao*, Fig. 1a). The comparative length of the scape is compared to the length of the pedicel, measured along the outer lateral surface. ‘Flagellar style’ used by Lyneborg (2001) to describe the 2^nd^ and 3^rd^ flagellomeres is not used here, instead we use the latter terms. Epandrium length is measured along the midline, even though the epandrium of all species is apically emarginate. See Lyneborg (2001) for diagrammatic positions of morphological characters.

Terminalia of both male and female were macerated in 10% KOH at 50°C for one hour to dissolve soft tissue; neutralised with acetic acid, rinsed in distilled water, and then dissected in 80% ethanol. Female dissections were made to examine and illustrate sternite 8 and the furca. Preparations were placed in glycerol and figures drawn with the aid of a camera lucida mounted on a Zeiss Stemi SV 11 stereo-microscope. Genitalia preparations are stored in glycerol in a genitalia vial mounted on the pin beneath the specimen.

Collection Acronym: ANIC, Australian National Insect Collection, CSIRO National Facilities & Collections, Canberra, Australia; MEIC, M. E. Irwin Collection, Tucson, AZ, USA. Collection Database Numbers: ANIC_29:##### http://anic.ento.csiro.au/database/index.aspx; MEIC, Mandala Database; MEL_#####  http://www.inhs.illinois.edu/reasearch/mandala/

**Abbreviations**: *ab*, anterior beam; *ao*, anterior ocellus; *ap.s*, appendicular sacs; *avl*, anteroventral lobe; *c*, cercus; *d*, distiphallus; *da*, dorsal apodeme of parameral sheath; *dc*, dorsocentral macrosetae; *ea*, ejaculatory apodeme; *fca.* furca; *fg1*, 1^st^ flagellomere; *fg2*, 2^nd^ flagellomere; *fg3*, 3^rd^ flagellomere; *fw*, frons width; *ga*, gonocoxal apodeme; *gs*, gonostylus; *h*, hypandrium; *hp*, hypoproct; *igp*, inner gonocoxal process; *is*, internal strut; *lea*, lateral ejaculatory; *np*, notopleural macrosetae; *og*, outer gonocoxal process; *pa*, post alar macrosetae; *pd*, posterodorsal; *pf*, parafacial; *sa*, supra-alarmacrosetae; *sc*, scutellar macrosetae; *sep*, subepandrial plate; *spa*, spermatheca; *spl.s.d*, spermathecal sac duct; *spl.s*, spermathecal sac; *va*, ventral apodeme of parameral sheath; *vl*, ventral lobe.

## Taxon treatments

### Anabarhynchus oceanus

Ferguson & Yeates 2014 sp. n.

urn:lsid:zoobank.org:act:3C8185ED-891A-46BB-87AA-AD5D6861CFE7

#### Materials

- **Type status:** Holotype. **Occurrence:** catalogNumber: ANIC_29:032452; recordedBy: D.J. & R.L. Ferguson; individualCount: 1; sex: Male; **Location:** country: Australia; stateProvince: Victoria; verbatimLocality: Mallacoota, Betka Beach; verbatimLatitude: 37°35'11.12"S; verbatimLongitude: 149°44'16.65"E; **Event:** samplingProtocol: hand net; startDayOfYear: 27 Oct; endDayOfYear: 01 Nov; year: 2013; **Record Level:** institutionCode: ANIC; collectionCode: ANIC; basisOfRecord: pinned specimen, stainless steel pin dorsally through thorax.
- **Type status:** Paratype. **Occurrence:** catalogNumber: ANIC_29:032453; recordedBy: D.J. & R.L. Ferguson; individualCount: 1; sex: male; **Location:** country: Australia; stateProvince: Victoria; verbatimLocality: Mallacoota, Betka Beach; verbatimLatitude: 37°35'11.12"S; verbatimLongitude: 149°44'16.65"E; **Event:** samplingProtocol: hand net; startDayOfYear: 27 Oct; endDayOfYear: 1 Nov; year: 2013; **Record Level:** institutionCode: ANIC; collectionCode: ANIC; basisOfRecord: pinned specimen, stainless steel dorsally throught thorax.
- **Type status:** Paratype. **Occurrence:** catalogNumber: ANIC_29:032454; recordedBy: D.J. & R.L. Ferguson; individualCount: 1; sex: male; **Location:** country: Australia; stateProvince: Victoria; verbatimLocality: Mallacoota, Betka Beach; verbatimLatitude: 37°35'11.12"S; verbatimLongitude: 149°44'16.65"E; **Event:** samplingProtocol: hand net; startDayOfYear: 27 Oct; endDayOfYear: 1 Nov; year: 2013; **Record Level:** institutionCode: ANIC; collectionCode: ANIC; basisOfRecord: pinned specimen, stainless steel dorsally through thorax.
- **Type status:** Paratype. **Occurrence:** catalogNumber: ANIC_29:032455; recordedBy: D.J. & R.L. Ferguson; individualCount: 1; sex: male; **Location:** country: Australia; stateProvince: Victoria; verbatimLocality: Mallacoot, Betka Beach; verbatimLatitude: 37°35'11.12"S; verbatimLongitude: 149°44'16.65"E; **Event:** samplingProtocol: hand net; startDayOfYear: 27 Oct; endDayOfYear: 1 Nov; year: 2013; **Record Level:** institutionCode: ANIC; collectionCode: ANIC; basisOfRecord: pinned specimen, stainless steel dorsally through thorax.
- **Type status:** Paratype. **Occurrence:** catalogNumber: ANIC_29:032456; recordedBy: D.J. & R.L. Ferguson; individualCount: 1; sex: male; **Location:** country: Australia; stateProvince: Victoria; verbatimLocality: Mallacoota, Betka Beach; verbatimLatitude: 37°35'11.12"S; verbatimLongitude: 149°44'16.65"E; **Event:** samplingProtocol: hand net; startDayOfYear: 27 Nov; endDayOfYear: 1 Dec; year: 2012; **Record Level:** institutionCode: ANIC; collectionCode: ANIC; basisOfRecord: pinned specimen, stainless steel dorsally through thorax.
- **Type status:** Paratype. **Occurrence:** catalogNumber: ANIC_29:032457; recordedBy: D.J. & R.L. Ferguson; individualCount: 1; sex: male; **Location:** country: Australia; stateProvince: Victoria; verbatimLocality: Mallacoota, Betka Beach; verbatimLatitude: 37°35'11.12"S; verbatimLongitude: 149°44'16.65"E; **Event:** samplingProtocol: hand net; startDayOfYear: 27 Nov; endDayOfYear: 1 Dec; year: 2012; **Record Level:** institutionCode: ANIC; collectionCode: ANIC; basisOfRecord: pinned specimen, stainless steel dorsally through thorax.
- **Type status:** Paratype. **Occurrence:** catalogNumber: ANIC_29:032458; recordedBy: D.J. & R.L. Ferguson; individualCount: 1; sex: female; **Location:** country: Australia; stateProvince: Victoria; verbatimLocality: Mallacoota, Betka Beach; verbatimLatitude: 37°35'11.12"S; verbatimLongitude: 149°44'16.65"E; **Event:** samplingProtocol: hand net; startDayOfYear: 27 Nov; endDayOfYear: 1 Dec; year: 2012; **Record Level:** institutionCode: ANIC; collectionCode: ANIC; basisOfRecord: pinned specimen, stainless steel dorsally through thorax.
- **Type status:** Paratype. **Occurrence:** catalogNumber: ANIC_29:032459; recordedBy: D.J. & R.L. Ferguson; individualCount: 1; sex: female; **Location:** country: Australia; stateProvince: Victoria; verbatimLocality: Mallacoota, Betka Beach; verbatimLatitude: 37°35'11.12"S; verbatimLongitude: 149°44'16.65"E; **Event:** samplingProtocol: hand net; startDayOfYear: 27 Oct; endDayOfYear: 1 Nov; year: 2013; **Record Level:** institutionCode: ANIC; collectionCode: ANIC; basisOfRecord: Pinned specimen, stainless steel dorsally through thorax.
- **Type status:** Paratype. **Occurrence:** catalogNumber: ANIC_29:032460; recordedBy: D.J. & R.L. Ferguson; individualCount: 1; sex: female; **Location:** country: Australia; stateProvince: Victoria; verbatimLocality: Mallacoota, Betka Beach; verbatimLatitude: 37°35'11.12"S; verbatimLongitude: 149°44'16.65"E; **Event:** samplingProtocol: hand net; startDayOfYear: 27 Oct; endDayOfYear: 1 Nov; year: 2013; **Record Level:** institutionCode: ANIC; collectionCode: ANIC; basisOfRecord: pinned specimen, stainless steel dorsally through thorax.
- **Type status:** Paratype. **Occurrence:** catalogNumber: ANIC_29:032461; recordedBy: D.J. & R.L. Ferguson; individualCount: 1; sex: female; **Location:** country: Australia; stateProvince: Victoria; verbatimLocality: Mallacoota, Betka Beach; verbatimLatitude: 37°35'11.12"S; verbatimLongitude: 149°44'16.65"E; **Event:** samplingProtocol: hand net; startDayOfYear: 27 Oct; endDayOfYear: 1 Nov; year: 2013; **Record Level:** institutionCode: ANIC; collectionCode: ANIC; basisOfRecord: pinned specimen, stainless steel dorsally through thorax.
- **Type status:** Paratype. **Occurrence:** catalogNumber: ANIC_29:032462; recordedBy: D.J. & R.L. Ferguson; individualCount: 1; sex: female; **Location:** country: Australia; stateProvince: Victoria; verbatimLocality: Mallacoota, Beka Beach; verbatimLatitude: 37°35'11.12"S; verbatimLongitude: 149°44'16.65"E; **Event:** samplingProtocol: hand net; startDayOfYear: 27 Nov; endDayOfYear: 1 Dec; year: 2012; **Record Level:** institutionCode: ANIC; collectionCode: ANIC; basisOfRecord: pinned specimen, stainless steel dorsally through thorax.
- **Type status:** Paratype. **Occurrence:** catalogNumber: ANIC_29:032463; recordedBy: D.J. & R.L. Ferguson; individualCount: 1; sex: female; **Location:** country: Australia; stateProvince: Victoria; verbatimLocality: Mallacoota, Betka Beach; verbatimLatitude: 37°35'11.12"S; verbatimLongitude: 149°44'16.65"E; **Event:** samplingProtocol: hand net; startDayOfYear: 27 Oct; endDayOfYear: 1 Nov; year: 2013; **Record Level:** institutionCode: ANIC; collectionCode: ANIC; basisOfRecord: pinned specimen, stainless steel dorsally through thorax.
- **Type status:** Paratype. **Occurrence:** catalogNumber: ANIC_29:032464; recordedBy: D.J. & R.L. Ferguson; individualCount: 1; sex: female; **Location:** country: Australia; stateProvince: Victoria; verbatimLocality: Mallacoota, Betka Beach; verbatimLatitude: 37°35'11.12"S; verbatimLongitude: 149°44'16.65"E; **Event:** samplingProtocol: hand net; startDayOfYear: 27 Oct; endDayOfYear: 1 Nov; year: 2013; **Record Level:** institutionCode: ANIC; collectionCode: ANIC; basisOfRecord: pinned specimen, stainless steel dorsally through thorax.
- **Type status:** Paratype. **Occurrence:** catalogNumber: ANIC_29:032465; recordedBy: D.J. & R.L. Ferguson; individualCount: 1; sex: female; **Location:** country: Australia; stateProvince: Victoria; verbatimLocality: Mallacoota, Betka Beach; verbatimLatitude: 37°35'11.12"S; verbatimLongitude: 149°44'16.65"E; **Event:** samplingProtocol: hand net; startDayOfYear: 27 Oct; endDayOfYear: 1 Nov; year: 2013; **Record Level:** institutionCode: ANIC; collectionCode: ANIC; basisOfRecord: pinned specimen, stainless steel dorsally through thorax.
- **Type status:** Paratype. **Occurrence:** catalogNumber: ANIC_29:032466; recordedBy: D.J. &  R.L. Ferguson; individualCount: 1; sex: female; **Location:** country: Australia; stateProvince: Victoria; verbatimLocality: Mallacoota, Betka Beach; verbatimLatitude: 37°35'11.12"S; verbatimLongitude: 149°44'16.65"E; **Event:** samplingProtocol: hand net; startDayOfYear: 27 Oct; endDayOfYear: 1 Nov; year: 2013; **Record Level:** institutionCode: ANIC; collectionCode: ANIC; basisOfRecord: pinned specimen, stainless steel dorsally through thorax.
- **Type status:** Paratype. **Occurrence:** catalogNumber: ANIC_29:032467; recordedBy: D.J. & R.L. Ferguson; individualCount: 1; sex: female; **Location:** country: Australia; stateProvince: Victoria; verbatimLocality: Mallacoota, Betka Beach; verbatimLatitude: 37°35'11.12"S; verbatimLongitude: 149°44'16.65"E; **Event:** samplingProtocol: hand net; startDayOfYear: 27 Nov; endDayOfYear: 1 Dec; year: 2012; **Record Level:** institutionCode: ANIC; collectionCode: ANIC; basisOfRecord: pinned specimen, stainless steel dorsally through thorax.
- **Type status:** Paratype. **Occurrence:** catalogNumber: ANIC_29:032468; recordedBy: D.J. & R.L. Ferguson; individualCount: 1; sex: female; **Location:** country: Australia; stateProvince: Victoria; verbatimLocality: Mallacoota, Betka Beach; verbatimLatitude: 37°35'11.12"S; verbatimLongitude: 149°44'16.65"E; **Event:** samplingProtocol: hand net; startDayOfYear: 27 Nov; endDayOfYear: 1 Dec; year: 2012; **Record Level:** institutionCode: ANIC; collectionCode: ANIC; basisOfRecord: pinned specimen, stainless steel dorsally through thorax.
- **Type status:** Paratype. **Occurrence:** catalogNumber: ANIC_29:032469; recordedBy: D.J. & R.L. Ferguson; individualCount: 2; sex: male & female (in-coup); **Location:** country: Australia; stateProvince: Victoria; verbatimLocality: Mallacoota, Betka Beach; verbatimLatitude: 37°35'11.12"S; verbatimLongitude: 149°44'16.65"E; **Event:** samplingProtocol: hand net; startDayOfYear: 27 Nov; endDayOfYear: 1 Dec; year: 2012; **Record Level:** institutionCode: ANIC; collectionCode: ANIC; basisOfRecord: micro pinned laterally into pith block on single stainless steel pin.

#### Description

**Figure 2.**
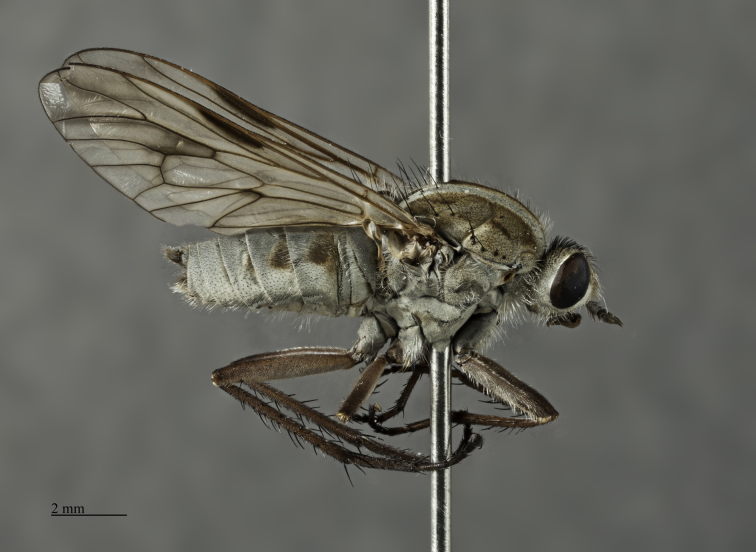
*Anabarhynchus oceanus* sp. n., Holotype male (ANIC_29: 032452): lateral view.

**Figure 3.**
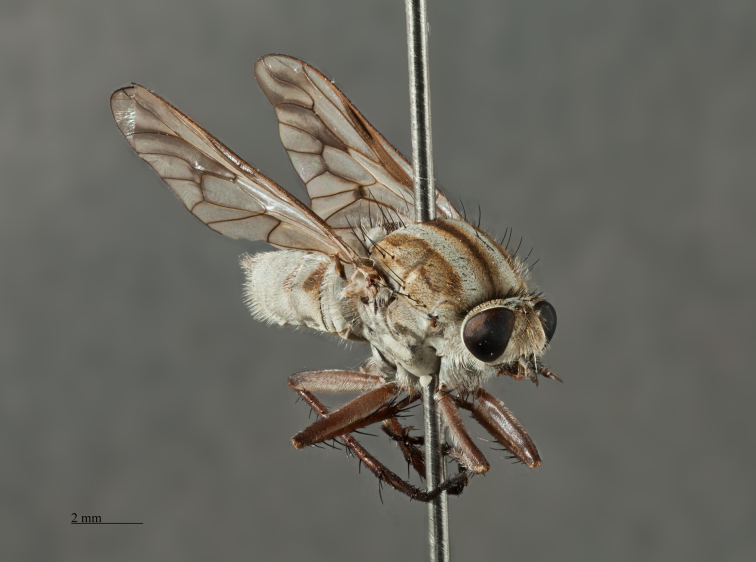
*Anabarhynchus oceanus* sp. n., Holotype male, (ANIC_29: 032452): oblique view.

**Figure 4.**
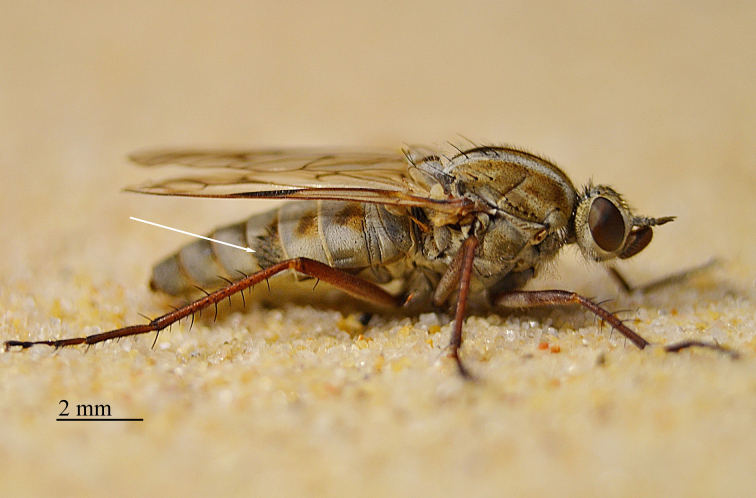
*Anabarhynchus oceanus* sp. n., female habitus; note patch of appressed black pile on tergite 4 indicated by white arrow [photo: D.J. Ferguson].

*Male*. Body Length: 11 mm; Wing Length: 11 mm; (Figs 2, 3, 4, 5) *Head*. Frons bulging; width at anterior ocellus 5.7 x ocellus; face and lower frons protruding; parafacial (*pf*, Fig. 1b) grey with sparse white pile; area lateral to antennal base with white pile; lower frons grey transitioning to yellow grey on mid and upper frons. Frontal pale brown, admixed with lesser amounts of erect dark pile, equal in length to scape. Scape and pedicel grey; scape 2.9 x pedicel length with strong black setae admixed with lesser pale on ventral surface; *fg1* blackish grey with short black setae around basal third; *fg2* & *fg3* black, one third length of *fg1*. Occiput convex with several indistinct rows of black macrosetae, pubescence yellowish grey; 47 setae on each side; postocciput area to gena grey with long, white, hair-like pile. Palp grey with long pale pile.

*Thorax*. Scutal chaetotaxy (pairs): *np*, 5; *sa*, 2; *pa*, 1; *dc*, 4-5; *sc*, 2, all black; Scutum with a pair of distinctive grey vittae on a pale brown ground, medially with a dark brown dorsal line that is finely tapered anteriorly; scutal surface with short pale, erect hairs on grey vittae and short black, erect hairs on dark brown dorsal line. Katepisternum with a few hairs dorsocentrally; prosternal furrow without pile; pleura grey; coxae grey with long, hair-like pile admixed with pale macrosetae; hind-coxa knob reduced. *Wing*. Slightly opaque, veins dark brown with brown infuscate along the basal *CuA1* vein, to the *m-cu* cross-vein; *M1+2*, distal of *r-m* cross-vein and along *R4+5* and *R5* veins with brown infuscate; stigma dark brown; costal setae beyond humeral cross-vein biserially arranged with the occasional additional singular setae outside rows. *Halter*. Pedicel blackish grey; knob pale brown ventrally, dark grey dorsally. *Legs*. Fore and middle femora without macrosetae; hind femur with 1-2 av macrosetae; all femora pale brown with grey pubescence; all tibia and tarsi pale-brown overlain with grey pubescence.

*Abdomen*. Integument black; all tergites covered with dense grey pubescence; lateral areas of tergites with dark brown blotches; surfaces covered with appressed and erect pale pile; sternites similar to tergites, without brown blotches. *Terminalia*. Paratype male (ANIC_29:032467): Epandrium (Fig. 5a), slightly longer than wide; tapered towards posterior, length approximately 2/3 width, anterior margin relatively flat, posterior margin emarginate medially; cercus tapered posteriorly with rounded apex, evenly distributed with pale setae; epandrium lateral edge with darkly sclerotised inwardly directed lobe, broadest anteriorly, tapered posteriorly; subepandrial plate, posterolateral corner with dark sclerotised, dorsoventrally folded process, forming a slightly concave anterior surface, ventral edge raised as a ridge. Gonocoxite rounded (Fig. 5b), with strong pale macrosetae; outer gonocoxal process posteriorly directed, slightly longer than wide; hypandrium strong, medially with broad triangular sclerite; gonocoxal apodeme darkly sclerotised. Inner gonocoxal process as long as outer gonocoxal process when viewed dorsally, strongly curved inward and tapered apically, a row of weak pale pile along dorsal surface of the apical half. Gonostylus directed inward and posteriorly, basal and medial dorsal surface with long pale setae directed inward and dorsally apex spatulas and reflexed dorsally. Ventral lobe darkly sclerotised, tapered to a sharp apex. Aedeagus (Fig. 5c); distiphallus ventrally directed; parameral sheath moderately sclerotised; ventral apodeme broadly flared, slightly projecting anteromedially, midline ventrally with thin flared ridge; lateral ejaculatory apodeme dark sclerotised; ejaculatory apodeme laterally flared, apical edge dark sclerotised.

**Variation.** Frontal pile variable ranging from all pale brown to nearly all black. Katepisternum pile varies from only several hairs on the dorsal area to none at all. *Male*. Body length: 11-11.5 mm. Wing length: 10.5 mm; wing range from relatively clear to slightly opaque. Frons width at anterior ocellar 5.1-6.1 x ocellar; occipital macrosetae 44-54 each side. *Female*. Body length: 12-13 mm. Wing length: 10.5-11 mm. Frons width at anterior ocellar 4.6-7 x ocellar; occipital macrosetae 34-42 each side; tergites 4 dorsolateral with a patch of long, appressed, black setae (white arrow, Fig. 4). *Terminalia*. Paratype female (ANIC_29:032468): sternite 8 (Fig. 5d): roundish in shape; anterior edge with broad cleft; anterior half darkly sclerotised, raised medially; patches of strong black setae along the posterolateral margin of raised area; weak pale setae distributed broadly medially, and on the posterior half of the dark sclerotised areas and marginally on the pale sclerotised regions; posterior mid-line poorly sclerotised; posterolaterally with broad pale areas tapering anteromedially. Furca (Fig. 5e): posterior frame weak; frame medially broad, dark sclerotised, lateral edge lobed and dorsally directed; anteroventral lobe large and broad and extending half-length of frame. Female internal organs (Fig. 5e): spermatheca (*spa*, Fig. 5f) subspherical; spermathecal sac duct (*spl.d*, Fig. 5f) long and narrow; apex enlarged, and connecting to large, subspherical, spermathecal sac (*spl.s*, Fig. 5f) and two appendicular sacs (*ap.s*, Fig. 5f).

#### Diagnosis

Frons bulging; frontal pile pale admixed with fewer black hairs, as long as scape, semi-erect; parafacial and area lateral to antennal base with pale pile. Scutum with a pair of distinctive grey vittae on a pale brown ground, medially with a dark brown dorsal line tapered towards anterior; coxal macrosetae pale. Female sternite 8 subspherical with broad pale areas posterolaterally and extending medially; frame of furca medially broad, anteroventral lobe large, extending half the length of the frame. Male epandrium with a modification to the subepandrial plate forming a dorsoventral wall towards posterolateral corner; ventral lobes of epandrium narrow and distinctively elongate.

#### Etymology

The species epithet ‘*oceanus*’ is derived from ocean beaches where the *type* specimens where collected.

## Discussion

This species is known only from a series of 22 specimens collected between October and early December on Betka Beach, eastern Victoria. Adults were also observed along other ocean beaches in the same area.

**Figure 6.**
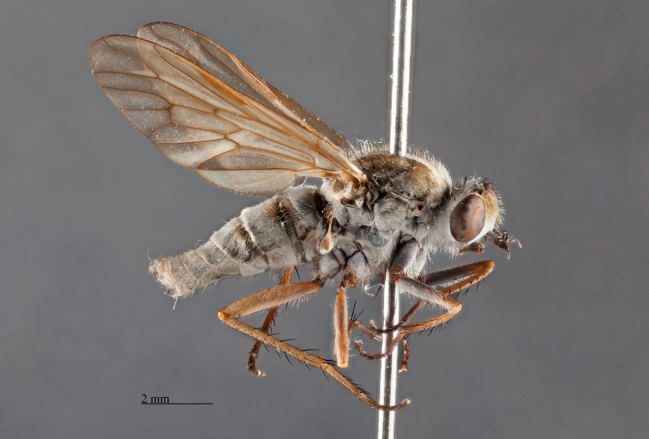
*Anabarhynchus kampmeierae* Irwin & Lyneborg (MEI_126727): male, lateral view.

*Anabarhynchus oceanus* sp. n. runs to couplet 4 in Lyneborg (2001) species key, however has pale pile on the parafacial, rather than black. The external morphology of *Anabarhynchus oceanus* sp. nov. is very similar to that of *Anabarhynchus kampmeierae* (Fig. 6) and could be misidentified, except for the following differences. The black postocular setae number 46-48 on each side (12-15 in *Anabarhynchus kampmeierae*); the fore femur is without macrosetae (1*pd* macroseta in *Anabarhynchus kampmeierae*); the katepisternum lacks or has only a few hairs on the dorsocentral area (numerous hairs on both ventral and dorsal surfaces in *Anabarhynchus kampmeierae*), and the prosternal furrow is without pile (with pile in *Anabarhynchus kampmeierae*). In the female, tergite 4 has a dense arrangement of appressed, long black hairs (Fig. 4; white arrow) that are lacking in *Anabarhynchus kampmeierae*.

The presence of white pile on the parafacial of *Anabarhynchus oceanus* sp. n. is consistent with the *Anabarhynchus kampmeierae* species-group. However *Anabarhynchus oceanus* sp. n. lacks a dome-shaped posterior lobe on the sternite 8, found in the *Anabarhynchus kampmeierae* group.

**Figure 7.**
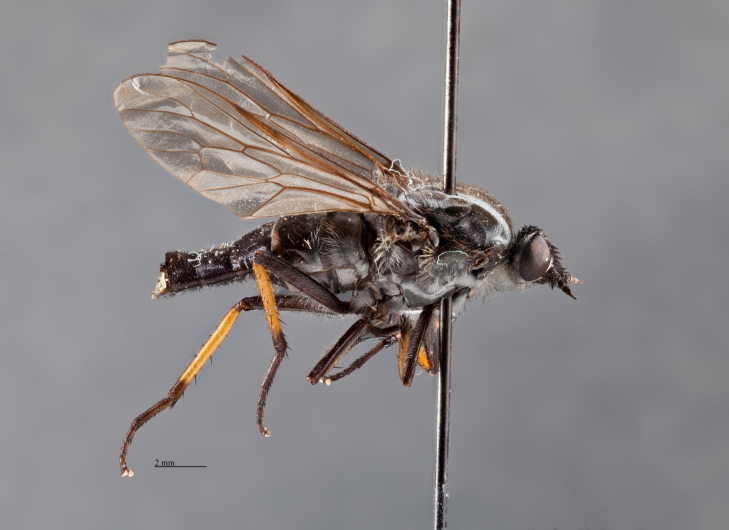
*Megathereva albopilosa* Lyneborg (MEI_156193): female, lateral view.

Our ongoing studies of *Anabarhynchus* show that new species do not fall neatly into the species groups defined by Lyneborg (2001). It is likely that species groups will need to be redefined as the fauna becomes better known. *Anabarhynchus oceanus* sp. n. appears to be most closely allied with the monobasic *Anabarhynchus kampmeierae* species-group as currently defined. *Anabarhynchus oceanus* sp. n. is also similar in habit to the related New Zealand genus *Megathereva* (Fig. 7) and shares a uniquely modified subepandrial plate with this genus. This may be evidence of an ancient Mesozoic Gondwanan connection between Australia and New Zealand, or evidence of more recent dispersal between Australia and New Zealand.  These two possibilities could be distinguished using molecular divergence time analyses as has been published recently by Lessard et al. (2013) for the scionine Tabanidae.

## Supplementary Material

XML Treatment for Anabarhynchus oceanus

## Figures and Tables

**Figure 1a.**
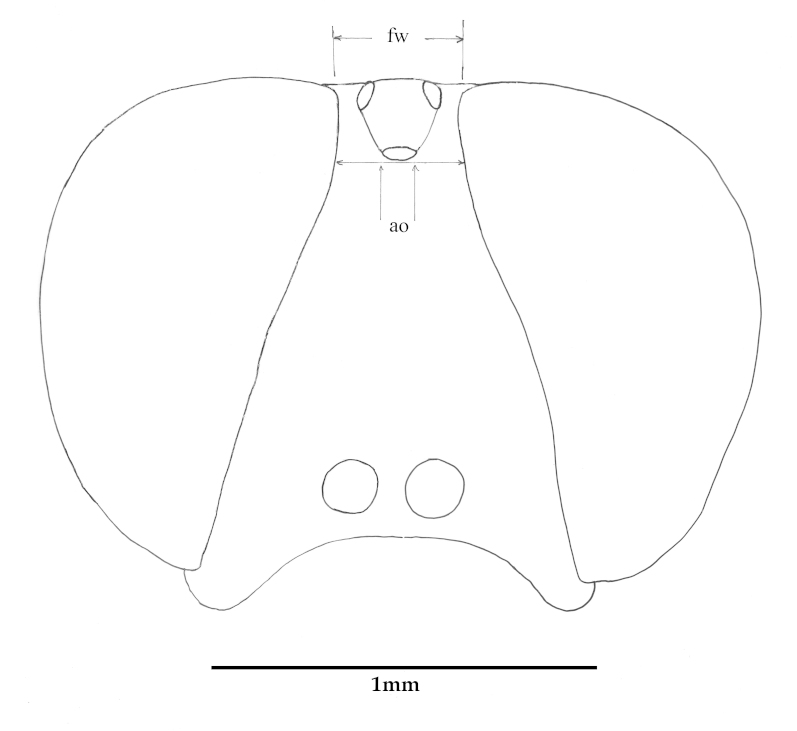
Frons width is determined by the width of the frons (fw) directly in front of the anterior ocellus, divided the width of the anterior ocellus (ao).

**Figure 1b.**
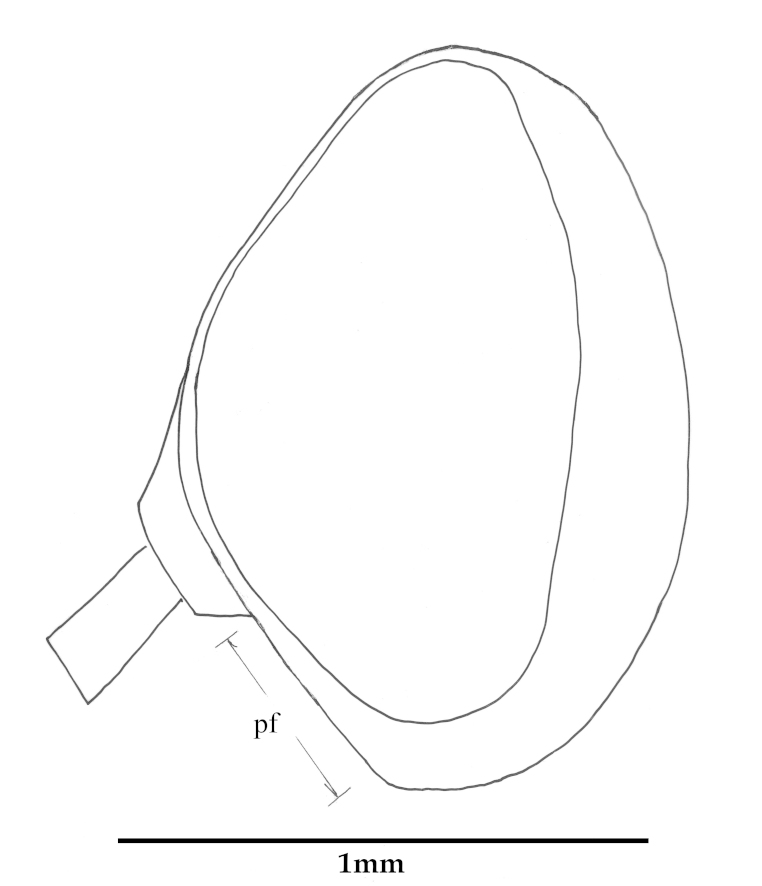
Indicating the position of parafacial (pf).

**Figure 5a.**
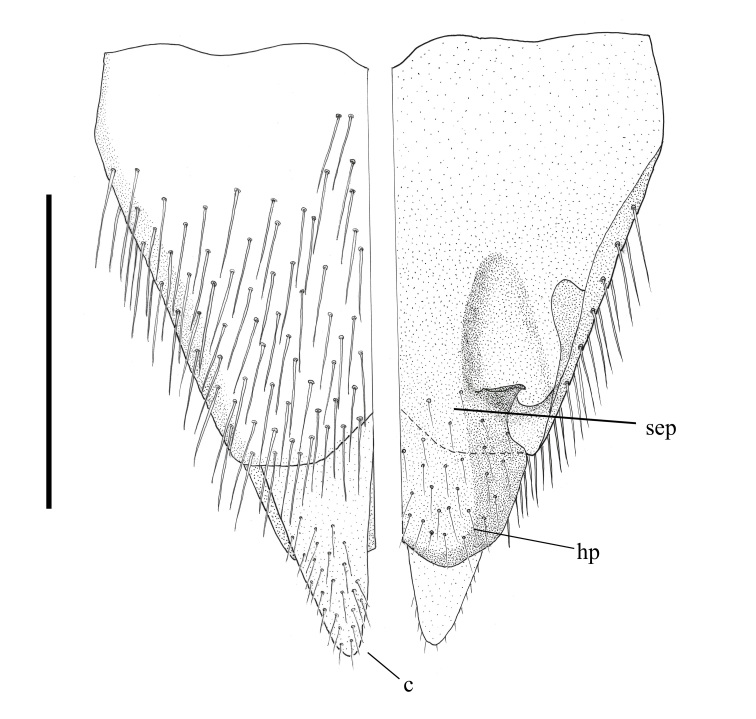
Epandrium, left dorsal view, right ventral view.

**Figure 5b.**
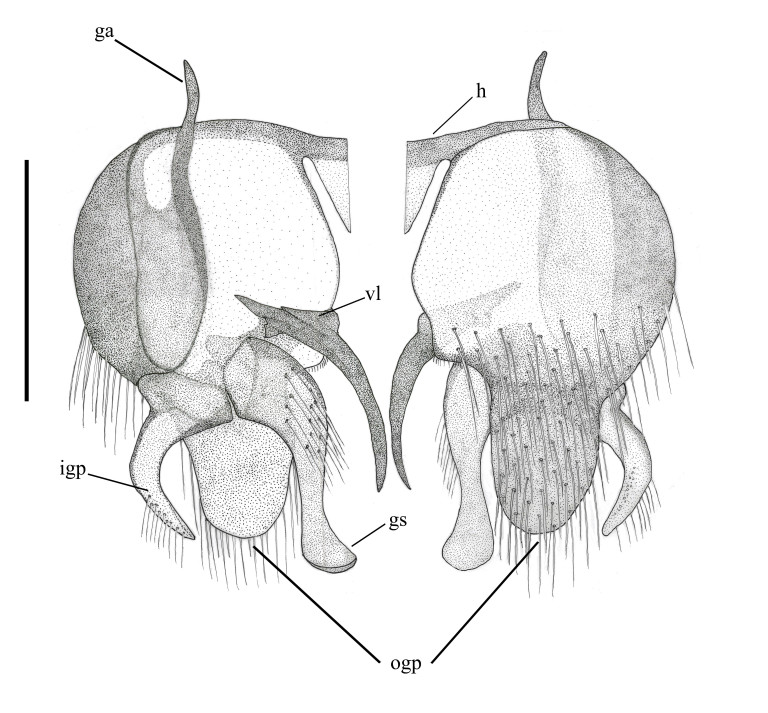
Gonocoxite, left dorsal view, right ventral view.

**Figure 5c.**
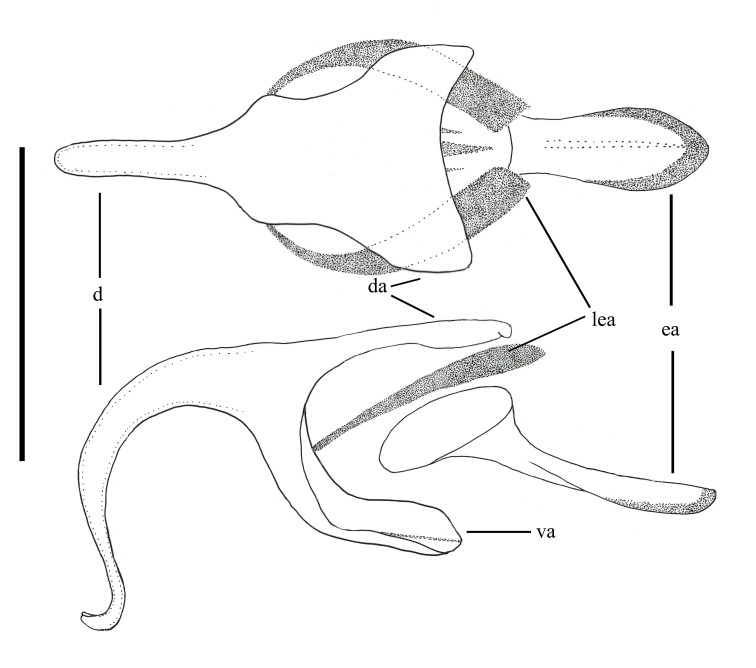
Aedeagus, dorsal view (upper), and lateral view (lower).

**Figure 5d.**
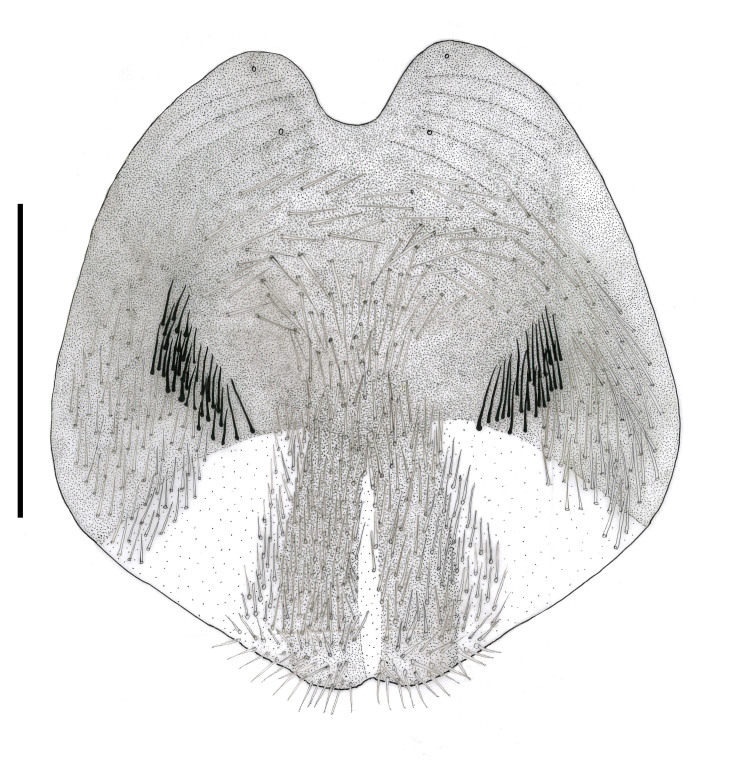
Female sternite 8, ventral surface.

**Figure 5e.**
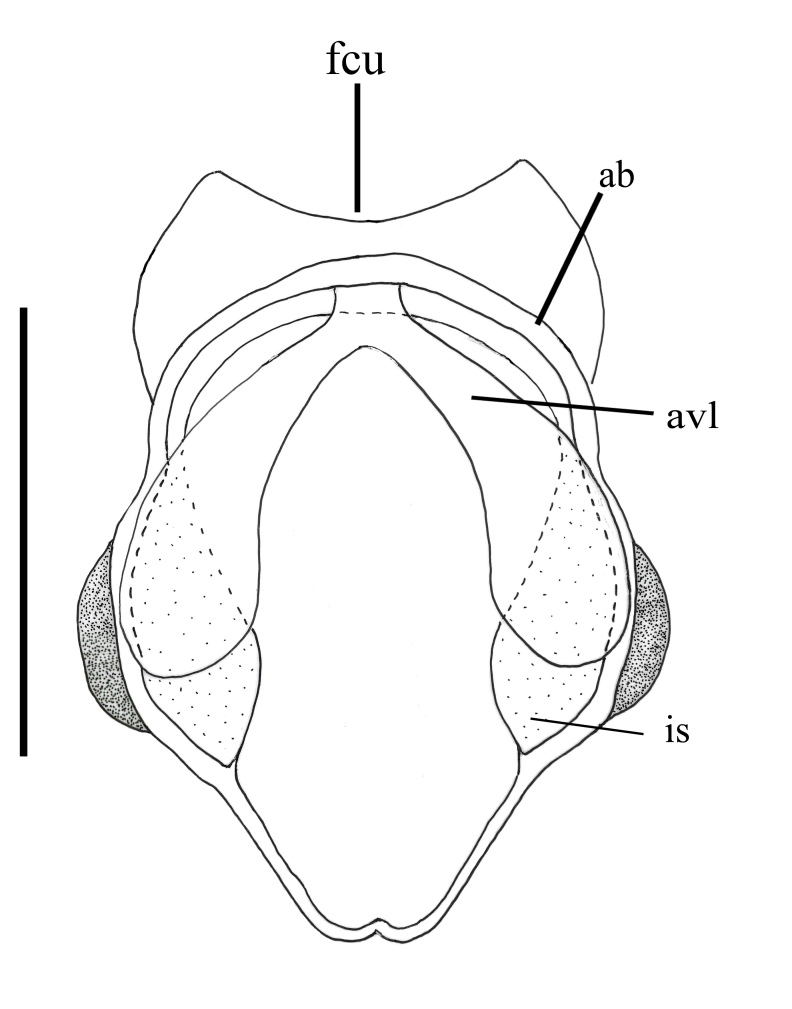
Female furca.

**Figure 5f.**
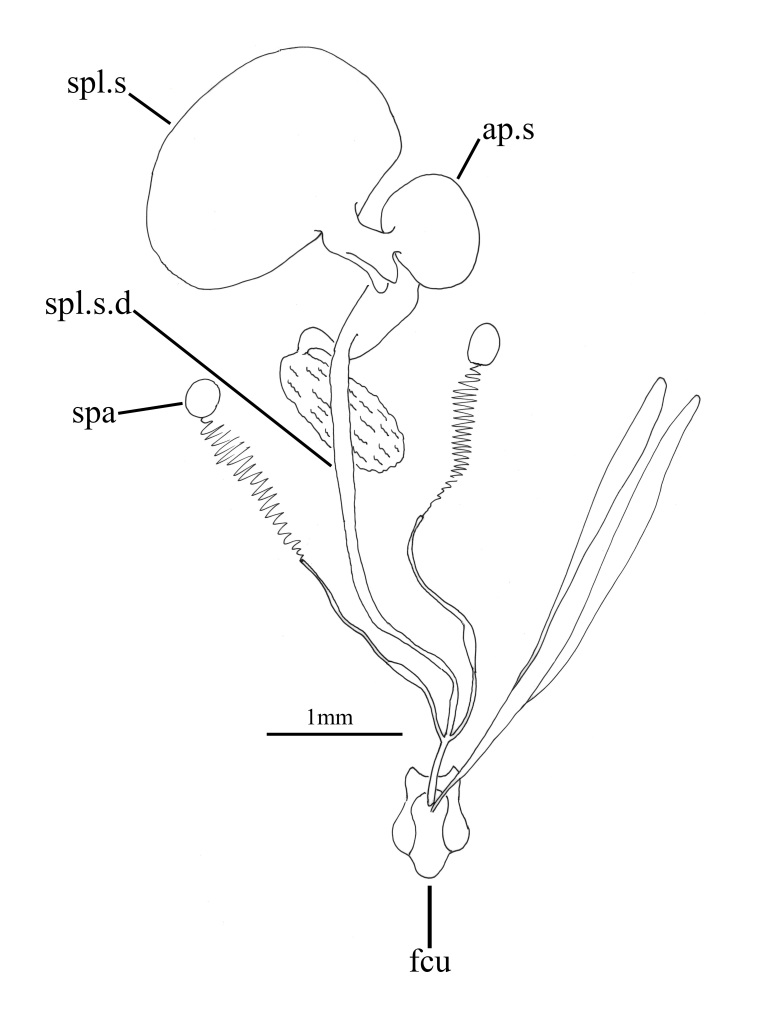
Female internal organs.
